# Patient connectivity with healthcare professionals and health insurer using digital health technologies during the COVID-19 pandemic: a German cross-sectional study

**DOI:** 10.1186/s12911-021-01605-8

**Published:** 2021-08-25

**Authors:** Niels Hannemann, Nina-Alexandra Götz, Lisa Schmidt, Ursula Hübner, Birgit Babitsch

**Affiliations:** 1 Department of New Public Health, Osnabrück University, Barbarastr. 22c, 49076 Osnabrück, Germany; 2 Health Informatics Research Group, Hochschule Osnabrück, Albrechtstr. 30, 49076 Osnabrück, Germany

**Keywords:** e-Health app, Digital divide, Health inequalities, COVID-19, Pandemic, Health literacy, Digital literacy

## Abstract

**Background:**

Digital health technologies enable patients to make a personal contribution to the improvement of their health by enabling them to manage their health. In order to exploit the potential of digital health technologies, Internet-based networking between patients and health care providers is required. However, this networking and access to digital health technologies are less prevalent in sociodemographically deprived cohorts. The paper explores how the use of digital health technologies, which connect patients with health care providers and health insurers has changed during the COVID-19 pandemic.

**Methods:**

The data from a German-based cross-sectional online study conducted between April 29 and May 8, 2020, were used for this purpose. A total of 1.570 participants were included in the study. Accordingly, the influence of sociodemographic determinants, subjective perceptions, and personal competencies will affect the use of online booking of medical appointments and medications, video consultations with providers, and the data transmission to health insurers via an app.

**Results:**

The highest level of education (OR 1.806) and the presence of a chronic illness (OR 1.706) particularly increased the likelihood of using online booking. With regard to data transmission via an app to a health insurance company, the strongest increase in the probability of use was shown by belonging to the highest subjective social status (OR 1.757) and generation Y (OR 2.303). Furthermore, the results show that the higher the subjectively perceived restriction of the subjects' life situation was due to the COVID-19 pandemic, the higher the relative probability of using online booking (OR 1.103) as well as data transmission via an app to a health insurance company (OR 1.113). In addition, higher digital literacy contributes to the use of online booking (OR 1.033) and data transmission via an app to the health insurer (OR 1.034).

**Conclusions:**

Socially determined differences can be identified for the likelihood of using digital technologies in health care, which persist even under restrictive conditions during the COVID-19 pandemic. Thus, the results indicate a digital divide with regard to the technologies investigated in this study.

## Background

E-health applications are considered as promising technologies that enable patients to positively participate in improving their state of health. These technologies support patients, e.g. in actively monitoring their own health condition and thereby participating in medical treatment and therapy decisions [1]. E-health encompasses all the apps that serve for the treatment and care of patients using modern information and communication media. As an overall term, it summarizes a broad spectrum of technological apps that process health information electronically, exchange it via secure connections between the actors involved, and thereby support the medical and therapeutic treatment processes [2]. As a subset of e-Health applications, mHealth supports health-related self-management by using mobile devices and health-related apps to monitor, measure, and analyze health-related data [3, 4].

E-Health and mHealth technologies are currently used in the context of individual health promotion, to support lifestyle changes, for the diagnosis and treatment of diseases, and for more efficient health care in structurally deprived and resource-poor regions. [5, 6]. For example, apps installed on smartphones and online-based video consultations offer patients the possibility of location-independent medical consultations as well as the exchange of health-related data with medical care providers [7]. However, it was found that the use of mHealth and health-related apps in Germany is below average: Only 28% of the inhabitants in Germany who have access to the Internet are using these kinds of apps to manage their health. Another 13% who have used health apps have stopped using them at some point [4, 8]. The results from a survey conducted in Germany between March and April 2020 shows that 33% of Germans have already used online booking for appointments [9]. Only 2% of the study participants [9] used an online-based video consultation with their doctor during the May survey period 2020, which was offered by every second outpatient care provider respectively 52% in May 2020 [10]. Moreover, the use of digital health technologies depends on the extent to which individuals and patients have sufficient technical infrastructure and access to the Internet [11]. The problem here is that access to the Internet and the availability of the necessary hardware are unevenly distributed in society. This situation is described by the term “digital divide” and refers to the fact that mostly socially deprived cohorts participate less in the digital transformation and benefit less from it [11–13].

In addition, as part of the ongoing processes of increasing digitalization, users are faced with the challenge of checking the personal and health-related relevance of information made available by digital health technologies to ensure that they can subsequently use the information in such a way that they are able to take responsibility for managing their own state of health and thereby contribute to an independent improvement in their individual state of health [8]. This means that, for example, simply owning a smartphone is not sufficient to ensure the adequate use of a health-related app and to be able to use digital health technologies appropriately and effectively [6]. In addition, a sufficiently high level of health literacy is required among users [6].

The exemplary results of previous studies show that the use of digital health technologies correlates with the level of health literacy and that high health literacy is associated with the more frequent use of Internet-based information searches on health-related topics and issues [14, 15]. Furthermore, health literacy varies by sociodemographic characteristics, such as age, migration status, and subjectively perceived social status, and to be less pronounced in the cohorts of lower socioeconomic status [16]. In particular, older people are considered to be an exemplary cohort who are less likely to use digital health technologies because they perceive their technical skills and their subjective assessment of digital competence to be insufficiently developed. [17]. Accordingly, it can be assumed that inequalities in access to digital health technologies will continue to increase and that the potential of digital health technologies to reduce such inequalities must be utilized and exploited [18, 19].

Since the outbreak of COVID-19 at the turn of 2019/2020, more than 170 million people worldwide in over 190 countries were infected with the COVID-19 virus, with more than 3.5 million deaths reported by the end of May 2021 [20, 21]. In Germany, more than 3.6 million confirmed infections with COVID-19 occurred, resulting in more than 86.000 deaths [22]. Almost all areas of life are affected by the impact of the COVID-19 pandemic [23]. For example, the care of COVID-19 patients involves a high expenditure of medical, material, and human resources, reflected by the incidence that treatment capacities for COVID-19 patients were reserved by canceling or postponing elective surgeries and preventive examinations to provide necessary intensive care treatment capacities for COVID-19 patients [24]. In addition to the expenditure of infrastructural and intensive care resources, healthcare professionals suffer from the physical and psychological stress associated with caring for COVID-19 patients, manifested in depression, distress, and subjectively poor perceived health [25].

Pandemic-related impacts arising outside the health care system include the fear of an infection with COVID-19 and dying from COVID-19 in the general population, as well as feelings of helplessness and depression due to social distancing and isolation, and concerns about getting fired because of the significantly decreased national economy and thus being unable to provide a living for themselves or their families [26–29]. In education, school and university closures occurred to contain the spread of the virus, including a transition to web-based lectures and homeschooling [30, 31].

Moreover, social epidemiological studies from the United States and the United Kingdom suggest that the risk of being infected with COVID-19, experiencing a severe illness course, or dying from the virus is more pronounced in socially or socioeconomically deprived populations compared to cohorts of higher socioeconomic status [32]. In addition, as regards the higher risk of contracting the disease when socioeconomic stratification is taken into consideration [32], the elderly are considered as a particularly vulnerable group of people during the COVID-19 pandemic [33, 34]. During the pandemic, the above-mentioned potentials of digital health technologies become apparent. Here, e-Health related technologies facilitate the exchange of treatment-relevant patient data between care providers or between patients and care providers by providing documents tailored to the individual needs of patients, thus enabling patient-centered care and treatment [35]. In combination with social distancing measures, these types of technologies open new ways to provide health services and support [36]. Digital health technologies / video consultations and conferences are suitable, for example, for location-independent health care and the monitoring of those who are chronically ill, for example, diabetics and pregnant women, since the risk of infection can be decreased, especially for these vulnerable cohort, by reducing contacts [36, 37]. Furthermore, digital health technologies can be used to disseminate trusted information about COVID-19 to the general population, which can improve people’s understanding of the disease and the provision of relevant healthcare services [38]. Some e-Health related technologies combine these functions by using an integrated tool for education, self-assessment, symptom monitoring and self-triage for COVID-19 for asymptomatic and symptomatic COVID-19 patients. It could be seen that unnecessary patient visits due to COIVD-19 were avoided to provide treatment capacities for emergency treatments and to contain the spread of the virus by the surveillance of COVID-19 infections supported by these kind of technologies [39, 40]. Nevertheless, Tebeje and Klein [35] concluded that most of present digital technologies which are used to overcome COVID-19 failed to provide information regarding cost-effectiveness and effectiveness.

Besides the potentials of these kinds of digital health technologies, there is the risk that the use of digital health technologies could generate new access barriers and inequalities. The elderly, in particular, are generally considered to be the population group with the poorest access to Internet-based technologies in this regard. [41]. The study by van Deursen [34] is an example of these barriers and inequalities regarding access to online-based information offerings about the virus in divergent social populations during COVID-19. The study investigated the information search on COVID-19-related information using the Internet during the COVID-19 pandemic. This study investigated the degree of need in the Dutch population to seek information about COVID-19 using the Internet and the influence of sociodemographic factors on COVID-19-related information seeking. The results showed that socioeconomically deprived as well as older cohorts are less able to use the Internet to obtain information about COVID-19-related topics than individuals with higher socioeconomic backgrounds. The relevant determinants here include lower levels of formal education, older age, poorer general literacy skills, and pre-existing physical infirmities. Therefore, the COVID-19 pandemic can be seen as reinforcing already existing digital inequalities [34]. Accordingly, during the COVID-19 pandemic, in particular, individuals need to be provided with evidence-based information about the virus so that they adopt preventive behaviors to counteract the fears about the virus and intentionally disseminated misinformation [42].

Therefore, it can be stated that pre-existing social and health inequalities are perpetuated in the digital setting and era and, accordingly, further empirical surveys are needed to better understand the influence of socio-demographic factors and health literacy on the use of digital health technologies. Furthermore, the current empirical research projects do not sufficiently consider the multicausal network of needs, attitudes, and reservations toward digital health technologies and the resulting target-group-specific and needs-based differentiation of eHealth technologies [11, 43].

Accordingly, this study pursues answering the question of whether or to what extent the use of digital health technologies has changed since the shutdown in Germany to control the COVID-19 pandemic and what influence divergent sociodemographic factors, subjective perceptions, and personal health literacy skills have on the use of digital health technologies, such as the online booking of medical appointments and medications, online-based video consultations with health care providers, and the transmission of health-related data via an app to health insurers.

The associated research hypotheses are as follows:

### Hypothesis No. 1

The use of online booking of medical appointments and medications, online-based video consultations with health care providers, and the transmission of health-related data via an app to health insurers changed during the COVID-19 pandemic compared to before the COVID-19 pandemic.

### Hypothesis No. 2

The younger the age group and the higher the socio-economic status, the more frequently the health technologies surveyed are used.

### Hypothesis No. 3

The use of the digital health technologies increases according to the subjectively perceived restriction of the life situation related to the COVID-19 pandemic.

### Hypothesis No. 4

The use of the digital health technologies increases according to the general health literacy, COVID-19-related health literacy, and digital literacy.

## Methods

### Study design

The results obtained are based on a partial analysis of the cross-sectional survey “Digital divide in relation to health literacy during the COVID-19 pandemic”. 7.239 people between 18- and 74-years old living in Germany were invited to complete an online questionnaire. A total of 1.953 people participated, and 1.570 individuals were included in the sample. The composition of the quota sample corresponds to the current distribution of age, gender, and residence in a federal state (not crossed) according to the Eurostat 2018 database. In the sample, the proportion of people with low education is larger than the national average. The study participants were recruited via Respondi AG, which is an external provider of online surveys. The external provider ensured anonymity and data protection guidelines in accordance with the General Data Protection Regulation (GDPR) at all times.

The data collection took place from April 29 to May 8, 2020. The timing of the study fell within the period when the first relaxations came into effect on April 20, 2020 after the shutdown of March 22, 2020 [44]. The questionnaire was broad in scope and captured the impact of the COVID-19 pandemic in different areas of life as well as health care, aspects of health, and as a focus, health literacy. For the purposes of this study, however, the focus will be on the use of digital health care technologies.

In the study period the numbers of infections were significantly lower with approximately 167.000 infected persons and 7.300 deaths [45] compared to the previously described situation at the time of submission in 2021. The study is a snapshot of the very specific circumstances regarding social life, COVID-19 exposure and burden as well as coping capabilities of the population and society.

### Measurement and operationalization

#### Use of digital health technologies before and since the shutdown to control the COVID-19-pandemic

The following three e-health services in outpatient care that are offered in Germany were examined: Use of online-based booking of doctor's appointments and medications, use of video consultation, and transmission of health-related data via an app to a health insurance company. The questions were closed-ended wherein the participants could answer either Yes or No. The study participants, who declared that they had already used a digital health service before or during the COVID-19 pandemic shutdown, were classified as users. All the items were combined into one score, with a range of values from 0 to 3.

### Analogous health care services in the ambulatory health care sector during COVID-19

The following variables were recorded under the general term analogous care situation in outpatient health care: Interruption of ongoing treatment due to the fear of a COVID-19 infection, difficulty in obtaining a medical appointment, cancellation of a medical appointment due to the fear of a COVID-19 infection, and cancellation of a medical appointment to protect relatives from a COVID-19 infection. The aforementioned variables depict care situations that occurred in outpatient analog medical care during the COVID-19 pandemic shutdown, thereby potentially influencing the use of digital health technologies. The aforementioned variables were recorded during the study on a four-point Likert scale (1 = *Do not agree at all* to 4 = Fully agree). The response categories *Do not agree at all* and *Rather does not agree* were recoded into the variable *Not appeared*. The response categories *Fully Agree* and *Rather agree* were recoded into the category *Appeared*.

### Sociodemographics

The following sociodemographic factors were included: Age, gender, migration status, education,, and subjective social status (SSS).

In contrast to the usual classification, a distinction is made here between the age groups based on generations in order to identify potential generational differences / effects regarding the usage of these digital health technologies [46]. The age generations are composed as follows: Traditionalists (born 1922–1955); Baby Boomers (born 1956–1965); Generation X (born 1966–1980); Generation Y (born 1981–1995); Generation Z (born 1996 and later). The educational level was collected using the CASMIN educational classification system [47]. It was followed by categorization into low, medium, and high education groups [48]. SSS was collected based on self-assessment. This was done by individual assignment on a ladder with levels from 1 (lowest social status) to 10 (highest social status) [49]. Following Höbel et al. [50], the study participants were assigned a low (scale values 1–4), medium (scale values 5–6), and high (scale values 7–10) SSS.

The classification into the migration status category followed the recommendation of Schenk et al. [51]. A migration status exists if both parents were born in another country or the respondent has not lived in Germany since birth and at least one parent was born abroad, or their native language is not German.

The residence was categorized as follows: Rural area (up to 5.000 inhabitants); Small town (5.001–20.000 inhabitants); Medium-sized city (20.001–100.000 inhabitants); Urban city (more than 100.000 inhabitants) [52]

### Health-related variables, Chronic illness

The operationalization of the variable subjective health was based on the subjective assessment (poor, less good, satisfactory, good to very good) of the personal health status [53]. For the subsequent analysis, two categories were formed, which were operationalized as follows: less good/bad; good/very good health. In addition, the study participants were asked whether they were suffering from a chronic illness. The answer was given here with the help of the dichotomous expressions of yes or no.

### Subjectively perceived restriction of the life situation due to COVID-19

The participants were asked about their subjective perception of the COVID-19 pandemic and the extent to which they felt burdened, threatened, or restricted by COVID-19, which they indicated on a five-point Likert scale (1 = not at all to 5 = very much).

From these three items (Threat, Burden, Restriction due to COVID-19), a sum score was formed for the subjectively perceived restriction of the life situation, which could assume values between 3 and 15. Reliability analysis using Cronbach's alpha yielded a value of 0.793, which represents a sufficiently high inter-item correlation. This scale was designed as part of this study. The exclusion of any variable in the reliability analysis did not indicate an increase (Cronbach's alpha 0.600–0.784) of the inter-item correlation.

### General health literacy, COVID-19-related health literacy, and digital literacy

The general health literacy (GHL) was determined by a sum of six items on a five-point Likert scale (5 = *very easy* to 1 = *very difficult*), referring to Schaeffer et al. 2016 [54]. Therefore, the score can assume values between 6 and 30. A higher score indicates a higher GHL. Reliability analysis by Cronbach's alpha yielded a value of 0.895 and was, therefore, a sufficiently high internal consistency. COVID-19-related health literacy (COV-19-HL) was measured in the form of a scale sum value based on 10 items on a five-point Likert scale (1 = *Does not apply at all* to 5 = *Applies completely*). Conceptually, the measurement follows the recommendations of Okan et al. [55]. Therefore, a score between a minimum of 10 and a maximum of 50 could be achieved. Accordingly, a higher score here also means better COV-19-HL. The reliability analysis using Cronbach's alpha yielded a value of 0.830. The digital literacy (DL) was also formed with the use of a sum score in accordance to [56, 57] as a new scale for measuring the digital competence, which comprises the response options of a five-point Likert scale (1 = *Don't agree at all* to 5 = *Agree completely*) [56, 57]. Consequently, the sum score can assume values between 11 and 55. Reliability analysis using Cronbach's alpha yielded a value of 0.893, which also represents a sufficiently high inter-item correlation. Here, the scale only focuses on the competencies "Information and media literacy" and "Digital problem solving". The competencies "Communication and Collaboration" and "Content Creation" were not operationalized in this study. Moreover, the assessment of digital literacy does not only include the Internet, but also digital media as a whole.

### Data analysis

The statistical analysis included a descriptive presentation of the sample, a before-and-after comparison, and bivariate as well as multivariate procedures. The preparation of the data set and the subsequent data analysis were carried out with IBM SPSS 26.

The analysis of the before-after comparison of the dichotomous characteristic values regarding the use of the listed digital health technologies, based on the McNemar test, serves to test research hypothesis No. 1. Furthermore, an additional bivariate analysis is performed to test whether the extent of the outpatient analog care situation influences the use of the online-based booking of doctor appointments and medications, use of a video consultation, and transmission of patient-relevant data via an app to a health insurance company during the COVID-19 pandemic. To test research hypothesis No. 2, a bivariate analysis is conducted regarding the use of digital health services. The hypothesis was tested with the help of the stratification of sociodemographic characteristics. For the analysis, the variables of education level (CASMIN education classification system), SSS, subjective health status, chronic illness, gender, age generations, and migration status were enrolled. Since the requirements for parametric tests were not completely fulfilled, non-parametric test procedures (Kruskal–Wallis test/Wilcoxon-Mann–Whitney test) were used. To test research hypotheses No. 3 and No. 4, a block wise and inclusion-based binary logistic regression analysis is performed to determine the influence of sociodemographic factors, subjective feelings, and personal health literacy, in addition to the relative probability of using the addressed digital health technologies during the COVID-19 pandemic. Because of the low uptake of video consultation, this type of regression analysis was not performed as part of the multivariate analysis.

Previous results of an earlier conducted survey [34] indicate that the seeking of digital information during COVID-19 is associated with socioeconomic and sociodemographic factors. Consequently, the first block of the model primarily includes sociodemographic influencing factors. In the second step, the model was expanded to include the variable of subjectively perceived restriction experienced by the study participants in the wake of the COVID-19 pandemic. Considering the previous blocks and their associated variables, GHL, COV-19-HL, and DL were included in the third model. In addition, in each of the following regression models, the reference groups are consistent and categorized as follows:

- Gender (reference: male)
- Age group (reference: traditionalists)
- Migration status (reference: migration background existing)
- Education (reference: low formal education)
- Chronic illness (reference: no chronic illness)
- Subjective social status (reference: low SSS)
- Subjective health status (reference: less good/poor health)
- Residence (reference: rural area)

## Results

### Description of the sample

A total of 1.570 participants (see Table 1) responded to the online survey, including 785 women and 785 men. Generation X is most frequently represented in this sample with 27.8%. 8.4% of the test participants have a migration background. 41.5% of the participants classify themselves as having a medium SSS. In the education category, the medium level of education predominates with 51.7%; almost every second participant (47.2%) suffers from a chronic illness. At the same time, 62.2% of the study participants stated that they assessed their subjective health status as good to very good. Most of the participants (67.1%) do not have a residence in an urban city.

**Table 1:** Descriptive description of the sample

Characteristics	n
Gender	
Female	785 (50%)
Male	785 (50%)
Age generation	
Generation Z	163 (10.4%)
Generation Y	416 (26.5%)
Generation X	436 (27.7%)
Baby Boomer	345 (22%)
Traditionalists	210 (13.4%)
Migration background	132 (8.4%)
Subjective social status	
Low	370 (23.6%)
Medium	651 (41.5%)
High	549 (35%)
Education	
Low	476 (30.3%)
Medium	812 (51.7%)
High	282 (18%)
Chronic illness	741 (47.2%)
Subjective health status	
Poor to less good	155 (9.9%)
Satisfactory	439 (28%)
Good to very good	976 (62.2%)
Residence	
Rural area	330 (21.0%)
Small town	353 (22.5%)
Medium-sized city	370 (23.6%)
Urban city	517 (32.9%)

### Use of digital health services before and since the shutdown to contain the COVID-19 pandemic

**Fig. 1:**
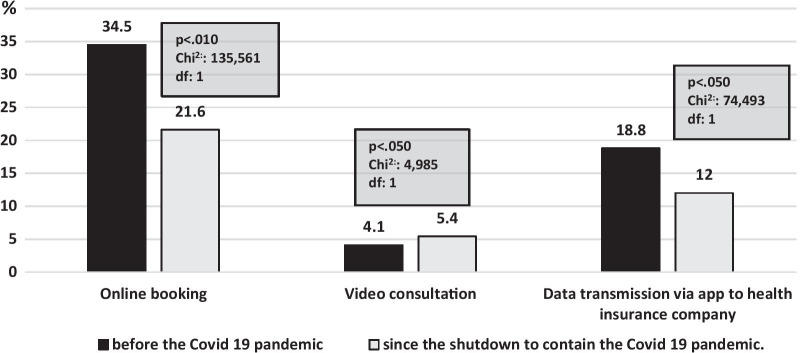
Use of online booking, video consultation, and data transmission via an app to a health insurance company before and since the shutdown to contain COVID-19

Before the COVID-19 outbreak, online booking was the most commonly used service at 34.5%. A total of 18.8% of respondents reported that they had already used an app to transmit data about health-related information to their health insurer. In comparison, the respondents were less likely to use a video consultation at 4.1% (see Fig. 1). The study showed that online booking was still used most frequently (21.6%) and video consultations the least frequently (5.4%) by the study participants. With regard to the use of online booking and the digital transmission of health-related data via an app to a health insurance company, a significant decrease was observed. On the other hand, a significant increase (1.3%) in the use of video consultations was found.

### Use of digital health technologies and the utilization of analog health care services since the shutdown to contain the COVID-19 pandemic

Overall, it appears that, despite the influence of the COVID 19 pandemic, the majority of participants did not stop using analog care services in ambulatory health care and, therefore, they were less likely to use the aforementioned digital health technologies (see Table 2).

**Table 2:** Analogous health care services in the ambulatory health care sector during the COVID-19 pandemic

Interruption of ongoing medical treatment because of the fear of a COVID-19 infection	Appeared	Not appeared	Wilcoxon-Mann–Whitney test
Number of health technologies used			
0	55.6%	71.2%	
1	24.2%	21.1%	
2	12.5%	5.8%	
3	7.7%	1.9%	*p* <0.001
Total (n = 1.036)	248	788	
Problems arranging a doctor's appointment			
0	54.1%	71.6%	
1	28.6%	20.3%	
2	9.8%	6.2%	
3	7.5%	1.9%	*p* <0.001
Total (n = 1.009)	266	743	
Cancellation of a doctor's appointment due to the fear of a COVID-19 infection			
0	59.8%	70.9%	
1	23.2%	21.1%	
2	9%	6.8%	
3	8%	1.2%	*p* <0.001
Total (n = 1.174)	311	863	
Cancellation of a medical appointment to protect relatives from a COVID-19 infection			
0	63.9%	70.1%	
1	23%	20.8%	
2	8%	7.5%	
3	5.2%	1.5%	*p* <0.050
Total (n = 1.157)	427	730	

55.6% of the participants who canceled their medical treatment due to the fear of a COVID-19 infection did not use any of the named digital health technologies. A subsequent significance test showed that the use of digital health technologies was significantly higher (*p* < 0.001) in the cohort that canceled treatment than in the cohort that did not. To the extent that problems were encountered in making appointments, more than half (54.1%) did not use any of the technologies mentioned. The subsequent significance test revealed a significantly higher use (*p* < 0.001) of digital health technologies among those subjects who had problems making a medical appointment compared with the cohort who had no problems. 59.8% of the participants who canceled their medical appointment because of their fear of COVID-19 infection did not use a digital health technology. The following interferential statistical test was able to attest to significantly higher use (*p* < 0.001) in the cohort who canceled the appointment than the cohort who did not. 63.9% of the respondents who canceled their scheduled medical appointment to protect loved ones from a COVID-19 infection did not use digital health technology. Again, the interference statistical test showed that, in the cohort of canceling an appointment, digital health technology use was significantly higher (*p* <0.050) compared with the individuals who attended the appointment anyway.

### Sociodemographic differences regarding the use of booking, video consultations, and the transmission of health-related data via an app to health insurers

Regarding the use of digital health technologies, the results show that with the increase of age or belonging to an older generation, the use of digital health technologies decreases. The following interferential statistical test showed that the use of digital health technologies was significantly higher in Generation Z compared to all the other groups (see Table 3). The use of digital health technologies is the highest among subjects with a higher SSS compared to the two other SSS groups. It was found that the use of digital health technologies by respondents from rural areas was significantly lower than in an urban and medium-sized city. In addition, the use of digital health technologies was significantly lower in a small town compared to a medium-sized city. Similar and significant results can also be found for education: the higher the level of education was, the higher the use of the aforementioned health technologies. Participants with a chronic illness used digital health technologies significantly more often (M_Rank_813.03) than healthy participants (M_Rank_760.89). Participants with migration background used digital health technologies significant more frequently (M_Rank_851.75) than subjects without a migration background (M_Rank_779.42). No significant gender differences were identified.

**Table 3:** Use of online booking, video consultation, and data transmission via an app to a health insurance company during the shutdown to contain COVID-19

Age generation	Generation Z	Generation Y	Generation X	Baby Boomer	Traditionalists	Kruskal–Wallis test
Number of health technologies used						
0	59.5%	69%	76.6%	74.2%	74.3%	
1	25.2%	20.2%	16.5%	19.7%	19.5%	
2	9.8%	7%	4.6%	5.5%	5.7%	
3	5.5%	3.8%	2.3%	0.6%	0.5%	*p* <0.001
Total (n = 1.570)	163	416	436	345	210	
Residence	Rural area	Small town	Medium-sized city	Urban city		Kruskal–Wallis test
Number of health technologies used						
0	76.1%	74.8%	68.6%	69.8%		
1	18.5%	16.4%	22.4%	20.1%		
2	4.5%	5.9%	6.8%	6.8%		
3	0.9%	2.8%	2.2%	3.3%		*p* <0.050
Total (n = 1.570)	330	353	370	517		
Subjective social status	Low	Medium	High			Kruskal–Wallis test
Number of health technologies used						
0	76.5%	72.2%	68.7%			
1	17.3%	19.8%	20.6%			
2	4.3%	6.2%	7.3%			
3	1.9%	1.8%	3.5%			*p* <0.050
Total (n = 1.570)	370	651	549			
Education	Low	Medium	High			
0	78.5%	71.4%	62.4%			
1	15.9%	20.1%	23.8%			
2	3.7%	6.7%	8.5%			
3	1.9%	1.7%	5.3%			*p* <0.001
Total	476	812	282			
Subjective health status	Good to very good	Poor to less good				Kruskal–Wallis test
0	72.2%	71.5%				
1	19.2%	20%				
2	5.6%	6.9%				
3	3%	1.5%				n.s
Total (n = 1.570)	976	594				
Gender	Male	Female				Wilcoxon-Mann–Whitney test
0	72.6%	71.3%				
1	18.5%	20.5%				
2	5.6%	6.6%				
3	3.3%	1.5%				n.s
Total (n = 1.570)	785	785				
Chronic illness	Not existing	Existing				Wilcoxon-Mann–Whitney test
0	75.2%	68.4%				
1	17.4%	21.9%				
2	4.6%	7.8%				
3	2.9%	1.9%				*p* <0.050
Total (n = 1.570)	829	741				
Migration background	No migration background	Migration background				Wilcoxon-Mann–Whitney test
0	72.6%	65.2%				
1	19.5%	18.9%				
2	5.8%	9.8%				
3	2.1%	6.1%				*p* <0.050
Total (n = 1.570)	1.438	132				

n.s. = not significant

### Logistic regression analysis: online booking of a doctor’s appointment / medication

The dependent variable in the following regression model is the use of an online booking of a doctor’s appointment. The following variables of model 1 have a significant influence on the probability of using an online booking of a doctor’s appointment in ranked order: High educational status, chronic illness, no migration status (see Table 4). In model 2, the variable no migration status was no longer found to be significant influencing factors. In addition, the variable subjectively perceived restriction of the life situation turns out to be a statistically significant influencing factor. In model 3, high SSS can no longer be identified as a significant influencing factor. In contrast, the variable “no migration status” was identified as a major influence factor, as in model 1. In addition, DL was identified as a significant variable influencing the probability of use. In model 3, a high educational status, chronic illness, and the subjectively perceived restriction of the life situation continue to exert a significant influence on the probability of use. Across all models, having the highest formal level of education (OR 2.119 [CI 1.454–3.087]; OR 2.122 [CI 1.454–3.097]; OR 1.806 [CI 1.228–2.657]) and having a chronic illness (OR 1.819 [CI 1.374–2.410]; OR 1.808 [CI 1.362–2.401]; OR 1.706 [CI 1.706–2.227]) significantly increased the likelihood of using online booking compared with the reference groups.

**Table 4:** Logistic regression Online **|** booking of a doctor’s appointment / medication

	Block 1 OR [CI 95%]	Block 2 OR [CI 95%]	Block 3 OR [CI 95%]
Variables			
*Gender (Ref. Male)*			
Female	0.970 [0.753–1.250]	0.933 [0.722–1.204]	0.966 [0.742–1.256]
Age Generation			
(Ref. traditionalists)			
Generation Z	1.631 [0.977–2.722]	1.536 [0.916–2.575]	1.591 [0.945–2,681]
Generation Y	0.944 [0.613–1.456]	0.886 [0.572–1.374]	0.884 [0.568–1.375]
Generation X	0.812 [0,533–1,239]	0.765 [0.499–1.172]	0.757 [0.492–1.164]
Baby Boomer	0.929 [0.608–1.419]	0.904 [0.590–1.386]	0.877 [0.570–1.350]
Education			
*(Ref. Low)*			
Medium	1.185 [0.864–1.625]	1.186 [0.864–1.628]	1.079 [0.782–1.490]
High	2.119 [1.454–3.087]**	2.122 [1.454–3.097]**	1.806 [1.228–2.657]*
Subjective social status			
*(Ref. Low)*			
Medium	1.295[0.926–1.810]	1.327 [0.947–1.859]	1.322 [0.940–1.858]
High	1.414 [0.995–2.010]	1.453 [1.020–2.069]*	1.353 [0.945–1.936]
Migration background			
*(Ref. Migration background existing)*			
No migration status	0.652 [0.432–0.985]*	0.692 [0.457–1.048]	0.645 [0.423–0.982]*
Chronic illness			
*(Ref. No chronic illness)*			
Chronic illness	1.819 [1.374–2.410]**	1.808[1.362–2.401]**	1.706 [1.278–2.277]**
Subjective health status			
*(Ref. poor to less good)*			
Good to very good	0.983 [0.738–1.309]	1.081 [0.807–1.448]	0.988 [0.732–1.333]
Residence			
*(Ref. Rural area)*			
Small town	0.933 [0.627–1.387]	0.946 [0.635–1.410]	0.935 [0.625–1.397]
Medium-sized city	1.313 [0.900–1.914]	1.311 [0.897–1.914]	1.317 [0.897–1.932]
Urban city	1.361 [0.958–1.934]	1.347 [0.946–1.918]	1.336 [0.035–1.909]
Subjectively perceived restriction of the life situation		1.102 [1.051–1.155]**	1.103 [1.051–1.156]**
GHL			1.002 [0.970–1.035]
COV-19-HL			1.024 [0.997–1.051]
DL			1.035 [1.012–1.059]*
-2 Log-Likelihood	1581.972	1564.733	1539.253
Cox & Snel R^2^	0.035	0.046	0.061
Nagelkerkes R^2^	0.054	0.071	0.094
N	1.570	1.570	1.570

^*^p <0.050; **p <0.001

### Logistic regression: data transmission of health-related data via an app to a health insurance company

The dependent variable in the following regression model is the use of an app for the transmission of health-related data to a health insurance company. The following variables in model 1 have a significant impact on the likelihood of transmitting health-related information to health insurers via an app in ranked order: Generation Y, Generation Z, high educational status, SSS, chronic illness, medium educational status, and female gender (see Table 5). In model 2, the variables already significant in model 1 continued to be significant influencing factors. The inclusion of the variable subjectively perceived restriction of the life situation also proved to be a significant influencing factor. In model 3, the high educational status no longer appeared to be significant influencing factors compared with models 1 and 2. In model 3, the variable DL proved to be a significant influencing factor. The other significant influencing factors from model 1 and 2 were also found to be significant under the influence of the variables from model 3. The blockwise inclusion of the variables from computational models increased the coefficient of determination and thereby contributed to an improved model fit. Both Generation Y (OR 2.335 CI [1.297–4.203]; OR 2.271 [CI 1.254–4.114]; OR 2.303 [CI 1.268–4.185]) and Generation Z (OR 2.194 [CI 1.090–4.414]; OR 2.097 [CI 1.036–4.3245]; OR 2.212 [CI 1,268- 4.585]) showed a significantly increased relative probability of use compared to the reference group. It is notable that, under the influence of contextual health literacy, both mean and high educational status could no longer be identified as significant influencing factors.

**Table 5:** Logistic regression**|**Data transmission of health-related data via an app to a health insurance company

	Block 1 OR [CI 95%]	Block 2 OR [CI 95%]	Block 3 OR [CI 95%]
Variables			
*Gender (Ref. Male)*			
Female	0.605 [0.439–0.834]*	0.574 [0.415–0.794]*	0.588 [0.422–0.820]*
Age generation			
*(Ref. Traditionalists)*			
Generation Z	2.194 [1.090–4.414]*	2.097 [1.036–4.245]*	2.212 [1.088–4.499]*
Generation Y	2.335 [1.297–4.203]*	2.271 [1.254–4.114]*	2.303 [1.268–4.185]*
Generation X	1.463 [0.813–2.634]	1.410 [0.780–2.549]	1.415 [0.780–2.566]
Baby Boomer	1.128 [0.610–2.086]	1.129 [0.608–2.096]	1.085 [0.582–2.021]
Education			
*(Ref. Low)*			
Medium	1.655 [1.097–2.497]*	1.670 [1.106–2.523]*	1.551 [1.021–2.357]*
High	1.919 [1.161–3.172]*	1.929 [1.167–2.191]*	1.655 [0.992–2.761]
Subjective social status			
*(Ref.:Low)*			
Medium	1.463 [0.945–2.266]	1.522 [0.980–2.363]	1.532 [0.983–2.388]
High	1.807 [1.148–2.844]*	1.875 [1.188–2.960]*	1.757 [1.107–2.787]*
Migration background			
*(Ref. Migration background existing)*			
No migration status	1.090 [0.621–1.912]	1.180 [0.669–2.080]	1.111 [0.626–1.972]
Chronic illness			
*(Ref. No chronic illness)*			
Chronic illness	1.802 [1.267–2.562]*	1.801 [1.262–2.570]*	1.645 [1.145–2.363]*
Subjective health status			
*(Ref. poor to less good)*			
Good to very good	0.904 [0.630–1.296]	0.961 [0.692–1.440]	0.881 [0.604–1.285]
Residence			
*(Ref. Rural area)*			
Small town	1.312 [0.807–2.133]	1.341 [0.823–2.186]	1.326 [0.811–2.169]
Medium-sized city	1.356 [0.840–2.190]	1.343 [0.830–2.173]	1.330 [0.818–2.163]
Urban city	1.091 [0.685–1.736]	1.073 [0.673–1.711]	1.043 [0.652–1.669]
Subjectively perceived restriction of the life situation		1.114 [1.049–1.184]**	1.113 [1.047–1.182]*
GHL			1.016 [0.974–1.059]
COV-19-HL			1.025 [0.992–1.059]
DL			1.033 [1.004–1.064]*
-2 Log-Likelihood	1110.589	1098.348	1079.741
Cox & Snel R^2^	0.028	0.035	0.047
Nagelkerkes R^2^	0.053	0.068	0.089
N	1.570	1.570	1.570

^*^p <0.050; **p <0.001

## Discussion

### Key findings

In general, the analysis of the cross-sectional data indicates that no increase in the use of the selected digital health technologies could be identified. The results indicate that, before the onset of the COVID-19 pandemic, except for video consultation, the study participants were more likely to use online booking or data transmission to health insurance than during or since the shutdown to contain the COVID-19 pandemic. Regarding the use of online booking, our findings are in with the EPatient Survey [9] which shows that online booking was the most frequently used digital health technology in Germany. The limited use of online-based video consultations can probably be explained by the fact that this technology was not widely provided by health care professionals and, therefore, not available to the participants [10]. In addition to the aspect of infrastructural availability of the technologies mentioned, further empirical findings indicate that intrapersonal and interpersonal factors influence the use of digital technologies, such as a lack of understanding of the potential benefits of digital health technologies, concerns about the data security of personal health-related information, operating difficulties, and the lack of recommendations and advice from trustworthy sources and from family and friends [58]. Moreover, it appears that the use of digital health technologies was more frequent in the cohort that forwent analogous health care services due to COVID-19. Nonetheless, the impact of the COVID-19 pandemic on the delivery of analogous health care services does not appear to be of central and exclusive influence favoring a more frequent use of the selected digital health technologies. Despite the changing analogous health care services, there was relatively lower use of digital health technologies as a result of the pandemic. As a result, hypothesis No. 1 can be confirmed, in that the COVID-19 pandemic changed the use of the health technologies mentioned for this study, but in a different direction than expected.

The results of the bivariate analysis and binary logistic regression analyses confirm the influence of age, SSS, educational status, and suffering from a chronic illness on the use of the highlighted health technologies. A closer look at the bivariate analysis (see Table 2) shows that digital health technology use follows a sociodemographic or socioeconomic gradient, with more privileged cohorts using the technologies listed here more frequently. Furthermore, the results of the binary logistic regression analyses indicate being part of a younger age generation, a higher level of education, and a higher SSS. With regard to the use of data transmission to a health insurance company, it could be shown that the probability of use was increased by more than twice in Generation Y and Z compared to the traditionalists. In terms of the data transmission Generation Y and Generation Z were found to be significantly more likely to use the service. We also identified suffering a chronic illness as a significant influencing variable that increased the use and likelihood of an online booking of a doctor’s appointment and the transmission of health-related data via an app to a health insurance company. Consequently, hypothesis No. 2 can also be confirmed, particularly regarding data transmission via app to the health insurer. Therefore, the results obtained here are consistent with comparable surveys that also found more frequent use of digital health-related technologies among younger participants [34, 59, 60]. Furthermore, the results of the survey here indicate that the relative likelihood of use of data transmission was significantly lower for women compared to men, but this result contrasts and is not in clear agreement with the results of the bivariate analyses and findings of previously conducted studies. In these surveys, it was found that women tended to use digital health technologies more frequently than men [11, 59]. Heponiemi et al. [61] mention further potential reasons why deprived groups in particular are less likely to use digital technologies and more likely to receive in-person services. Online services seem to be more suitable for uncomplicated or general health problems, as these can be dealt with in a standardized way. In contrast, health-related problems in disadvantaged cohorts appear to be more complex, which cannot be captured and managed through online-based and standardized processes. This requires cross-sectoral and coordinated interventions by multidisciplinary service providers, which digital technologies cannot provide. A significant predictor was subjectively perceived restriction of life situation related to the COVID-19 pandemic. The results of the study show that, with the increase of the subjectively perceived restriction of the life situation by one point, the relative probability of using online booking and data transmission via an app to a health insurance company increased by 10.4% and 11.2%, respectively. As a result, hypothesis No. 3 can be confirmed. A higher subjectively perceived the restriction of life situation is associated with a higher likelihood of use. Increasing DL by one point significantly increases the probability of using online booking and data transmission via app by 3.3% and 3.4%, respectively. Based on the data available here, however, GHL and COV-19-HL can be identified as non-significant influencing factors. Therefore, hypothesis No. 4 can only be confirmed in terms of the DL. Therefore, the results between the study here and the findings described earlier are consistent with caveats.

### Limitations

In the case of the present online survey, it can be reasonably assumed that there may already be a bias in the fact that, on average, participants were more Internet- or technology-savy than the population they represent. In addition, the availability of online video consultations needs to be critically examined, as only slightly more than half of the health care providers offered such a consultation option. Accordingly, a distortion of the video consultation hours used due to a non-area-wide offer cannot be ruled out.

Furthermore, a differentiated consideration of the participants who have a migration status according to the definition here is necessary. Here, too, it can be reasonably assumed that there is a systematic bias in that the participants with a migration status were also more Internet- or technology-savvy on average than the cohort they represented, thereby not allowing a generalization regarding the overall population. It also cannot be ruled out that biases exist between subjectively self-assessed health literacy and actual health literacy and the possibility that participants over- or underestimated their level of competence.

## Conclusion

Based on the findings obtained here, it can be concluded that the selected digital health technologies can help to ensure access to health services for all social groups, which were restricted by COVID-19. At the same time, however, it is also clear to see a digital divide stratified by sociodemographic and socioeconomic determinants. Although the participants tend to be technology-savy, the use of these digital health technologies seems to be low across all population groups. While the COVID-19 pandemic appears to be an exceptionally strong influencing factor, the present results suggest that digital health technology use is related to external influencing factors that were not measured in this study. Future research projects should examine interpersonal and intrapersonal influencing factors in more detail to identify the reasons for the nonuse of digital health technologies, to derive interventions designed to potentially compensate for existing disparities, and to contribute to the development of health technology-related digital divide research.

## Data Availability

The data of the study are not publicly archived. It is only possible to grant access to the data in individual cases after consultation with the authors.

## Abbreviations

CI: Confidence interval
COV-19-HL: COVID-19-related health literacy
DL: Digital literacy
GHL: General health literacy
M_Rank_: Mean rank
n.s.: Not significant
OR: Odds ratio
Sig: Significance
SSS: Subjective social status
